# Weak gluteus maximus and weak iliopsoas with normal gluteus maximus: Two complementary new signs to diagnose lower limb functional weakness

**DOI:** 10.1002/brb3.3135

**Published:** 2023-06-27

**Authors:** Masahiro Sonoo, Takamichi Kanbayashi, Shunsuke Kobayashi, Hiromasa Matsuno, Takahiro Nakayama, Ichiro Imafuku, Tetsuo Ando, Toshio Fukutake

**Affiliations:** ^1^ Department of Neurology Teikyo University School of Medicine Tokyo Japan; ^2^ Department of Neurology Jikei University School of Medicine Tokyo Japan; ^3^ Department of Neurology Yokohama Rosai Hospital Yokohama Japan; ^4^ Department of Neurology Kameda Medical Center Kamogawa Japan

**Keywords:** functional neurological disorder, functional weakness, gluteus maximus, Hoover test, iliopsoas

## Abstract

**Background and purpose:**

The diagnosis of functional neurological disorder should be actively made based on the neurological signs. We described two new complementary signs to diagnose functional weakness of the lower limb, “weak gluteus maximus (weak GM)” and “weak Iliopsoas with normal gluteus maximus (weak iliopsoas with normal GM),” and tested their validity.

**Methods:**

The tests comprised Medical Research Council (MRC) examinations of the iliopsoas and GM in the supine position. We retrospectively enrolled patients with functional weakness (FW) or structural weakness (SW) who presented with weakness of either iliopsoas or GM, or both. Weak GM means that the MRC score of GM is 4 or less. Its complementary sign, weak ilopsoas with normal GM, means that the MRC score of ilopsoas is 4 or less, whereas that of GM is 5.

**Results:**

Thirty‐one patients with FW and 72 patients with SW were enrolled. The weak GM sign was positive in all 31 patients with FW and in 11 patients with SW, that is, 100% sensitivity and 85% specificity. Therefore, the complementary sign, weak iliopsoas with normal GM, was 100% specific for SW.

**Discussion:**

Although 100% should be discounted considering limitations of this study, these signs will likely be helpful in differentiating between FW and SW in the general neurology setting. Downward pressing of the lower limb to the bed in the supine position is interpreted by the patient as an active movement exerted with an effort and might be preferentially impaired in FW.

## Graphical abstract

New signs to diagnose functional weakness of the lower limbs are reported. The "Weak Gluteus Maximus" sign suggests functional weakness. The "Weak Iliopsoas with normal Gluteus Maximus" sign strongly suggests structural weakness

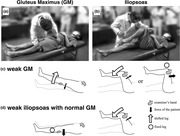

## 1 INTRODUCTION

Functional neurological disorder (FND) is a common disease (Stone, Carson, et al., 2010) that has been described from the ancient times (Weintraub, 1983). Conventionally, FND might have been diagnosed by exclusion or by identifying psychological causes, but experts have argued that it should be diagnosed by neurological signs themselves (Babinski, 1967; Stone et al., 2002; Weintraub, 1983). This opinion has been adopted by the newest Diagnostic and Statistical Manual of Mental Disorders, 5th edition, which required that “clinical findings provide evidence of incompatibility between the symptom and recognized neurological or medical conditions” for FND diagnosis (American Psychiatric Association, 2013). In other words, FND is now actively diagnosed based on “positive signs” that do not occur in structural neurological disorders, that is, by “rule‐in diagnosis” (Aybek & Perez, 2022; Daum et al., 2014; Perez et al., 2021; Stone, 2014; Stone et al., 2011; Weintraub, 1983).

Functional weakness (FW) is classified into the functional movement disorders, a subtype of FND (Hallett et al., 2022), and is one of the major signs of FND (Lanska, 2006; Stone et al., 2002; Stone, Warlow, et al., 2010). Many positive signs for FW have been reported (Aybek & Perez, 2022; Daum et al., 2015, 2014; Espay et al., 2018; Sonoo, 2023; Stone, 2014). The most famous one would be the Hoover test, (Hoover, 1908) which utilized a synergic movement of hip flexors and contralateral hip extensors (Sonoo, 2004). Because this test is apparently similar to the new signs presented in this study, we here would like to explain its details and supposed mechanism. In a healthy subject, the voluntary straight raise of one leg accompanies the contraction of not only the ipsilateral but also the contralateral psoas major muscle in similar degrees to stabilize the lumbar spine (Hu et al., 2011). The latter force needs to be balanced by the contraction of the contralateral gluteus maximus (GM) muscle. Accordingly, the whole set of these actions including the contractions of the ipsilateral iliopsoas and contralateral GM constitutes a synergic movement. The first author reported Sonoo abductor test (Lanska, 2006; Sonoo, 2004), which utilized a synergic movement of bilateral gluteus medius muscles. Both tests utilize the feature that functional paresis occurs “systematically” (Babinski, 1967). In the Hoover test, for instance, the whole synergic movement when the affected leg is raised is impaired in functional paresis, and therefore the GM muscle on the unaffected side also becomes weak. Similarly, the whole synergic movement when the unaffected leg is raised is normal, and therefore the GM muscle on the affected side also becomes strong.

Other positive signs of functional paresis include Babinski trunk‐thigh test (Babinski, 1967), global pattern of weakness, give‐way weakness (Magee, 1962), and many others (Aybek & Perez, 2022; Daum et al., 2015, 2014; Espay et al., 2018; Sonoo, 2023; Stone, 2014). However, these signs may not be validated, or insufficient specificities have been reported. For instance, give‐way weakness has been reported to be observed in one‐third of patients with structural weakness (SW), mainly stroke (Gould et al., 1986), although better specificities have been reported by other authors (Chabrol et al., 1995; Daum et al., 2015; Stone, Warlow, et al., 2010). The first author reported “paradoxical wrist flexion,” which described a phenomenon that cannot occur in structural paresis of the wrist flexor muscles (Sonoo, 2020).

One should not diagnose FND based on a single positive sign (Stone, Carson, et al., 2010), and therefore new positive signs or other neurological signs that help the differentiation between FND and structural neurological disorders (SND) are always of interest as their addition might increase the probability of correctly diagnosing FND. In this study, we investigated the utilities of a new sign, “weak gluteus maximus (weak GM),” and its complementary sign, “weak iliopsoas with normal gluteus maximus (weak iliopsoas with normal GM),” by retrospectively reviewing the medical records of patients presenting with FW or SW of the lower limbs.

## 2 MATERIALS AND METHODS

### 2.1 Subjects

The first author's out‐patient and EMG database and the records of the neurology ward round were retrospectively reviewed. The patients having the diagnostic keyword of FND, or those of SND that may cause lower‐limb weakness, including brain or spinal cord disorders, neuropathies or myopathies were consecutively extracted, and their medical records and EMG records, if present, were reviewed. Data extraction for the period from January to October of 2021 was performed for patients with FND and from April to October of 2021 for patients with SND. The inclusion criteria were as follows: (1) age was 20 or older; (2) the record was the first evaluation by the first author (Masahiro Sonoo); (3) neurological examinations including Medical Research Council (MRC) scores were conducted by the first author; (4) the MRC scores for the iliopsoas and GM were evaluated; (5) either, or both, iliopsoas or GM had a score of 4 or less in the weaker limb; and (6) the cause of the weakness was definitely established as either FND or a certain SND other than movement disorders such as Parkinson's disease (PD) or corticobasal syndrome (CBS), based on sufficient neurological examinations and appropriate ancillary tests. PD was excluded because of the reports of frequent associations of PD with FND (Hallett, 2018; Onofrj et al., 2011; Wissel et al., 2018), and CBS may present with signs mimicking functional weakness due to apraxia (Ercoli & Stone, 2020).

For the FW group, the followings were considered to be the signs to support the diagnosis of FW (supportive signs): (a) functional pattern in the Hoover test (Hoover, 1908), (b) functional pattern in the Sonoo abductor test (Sonoo, 2004), (c) paradoxical wrist flexion (Sonoo, 2020), (d) give‐way weakness, (e) normal and symmetrical reflexes despite asymmetrical weakness of the corresponding muscles, (f) other neurological signs suggesting FND such as typical functional gait (Lempert et al., 1991) and (g) normal recruitment with poor activation and no denervation potentials in needle EMG of a weak muscle. Based on these, another inclusion criterion for the FW group was set as follows: at least three of the above seven signs should be present. Other than these, Babinski sign was also evaluated, although it was not counted as a supportive sign. This is because Babinski sign may well be negative in neuropathies or myopathies as well as in some disorders with pyramidal signs such as amyotrophic lateral sclerosis (ALS) (Swash, 2012). When a structural disorder overlapped FW (functional overlay), the patient was included only when it was obvious that the structural disease did not affect the strengths of the hip joint muscles.

The retrospective study design was approved by the ethics committee of Teikyo University (approval number: 21–140), which approved the opt‐out method in obtaining the consent of patients.

### 2.2 MRC score evaluations

**FIGURE 1:**
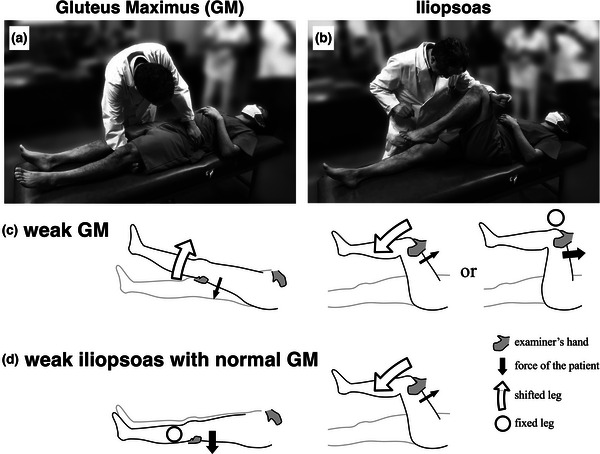
The maneuvers of the Medical Research Council (MRC) examinations for the gluteus maximus (GM) (a) and iliopsoas (b) (the examined subject is a staff of our department who is willing to cooperate with this study). The schemata of the two signs are also presented. When GM is weak with weak or normal iliopsoas (more frequently the former), “weak GM” sign is judged to be positive (c). When GM is strong and iliopsoas is weak, “weak iliopsoas with normal GM” sign is judged to be positive (d). Gray hand: examiner's hand. Black arrow: the force of the tested muscle of the patient. White large arrow: the tested leg which was shifted due to the examiner's force. White circle: the tested leg that was fixed against the examiner's force.

Only the MRC scores of the iliopsoas and GM muscles were considered in this study. The examinations were performed in the supine position. The test for the GM differed from the standard method in the prone position (Hislop et al., 2014; Kendall et al., 2005) but was similar to that in the supine position (Guarantors of Brain, 2010). However, our method (Figure 1a) was slightly different even from the latter in the textbook (Guarantors of Brain, 2010). The examiner's hand was placed under the knee of the examined lower‐limb. The patient was asked to strongly press down the lower‐limb toward the bed and to prevent it being raised from the bed by the examiner's hand under the knee. The examiner placed the other hand over the anterior superior iliac spine of the patient ipsilateral to the examined limb, and firmly fixed the pelvis toward the bed. In this way, the examiner could exert maximal force to elevate the lower‐limb, that is, to bend the hip joint. When the patient could not maintain this test position and the lower‐limb was lifted, the MRC score for the GM was judged to be 4 or less. We did not give a score of 3 since we did not perform the examination in the prone position. In any case, whether the MRC score was 5 or less than 5 was critical in this study.

For the iliopsoas, the hip joint was flexed at a right angle with the knee joint also flexed, and the patient was asked to maintain this position using maximal force against the examiner's hand (usually distal forearm) attempting to extend the hip joint (Figure 1b). When the patient could not maintain this test position and the hip joint was extended, the MRC score for the iliopsoas was judged to be 4 or less. Whether the MRC score of iliopsoas was 3 or less was judged by the examination in the sitting position, that is, we tested whether the patient can lift the thigh against gravity in the full range of motion.

We actually scored each muscle using a plus or minus suffix, for example, 4+ or 4−, but they were rounded to an integer number in this study. The MRC scores of only one leg for each patient were adopted, on the weaker side when the paresis was asymmetrical.

### 2.3 Evaluated parameters and the statistical analysis

**FIGURE 2:**
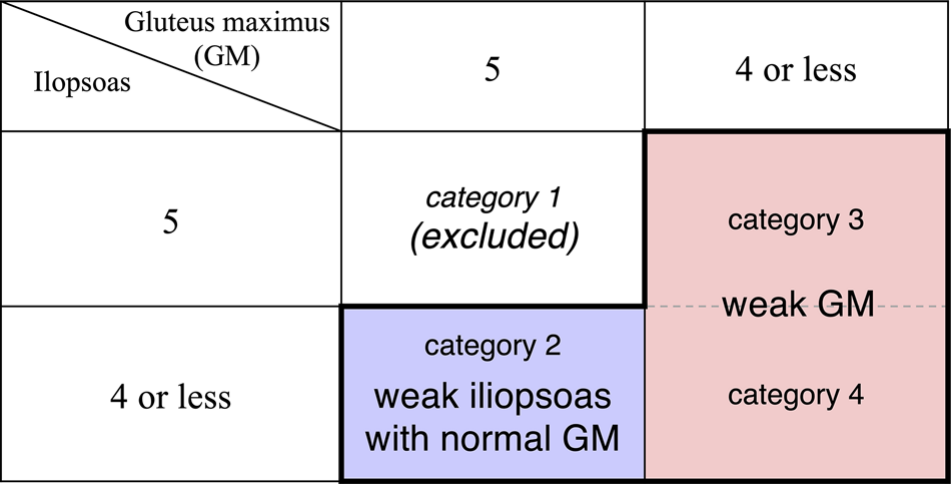
Classification of the main results. The area enclosed by a thick line represents the study population in this study. GM, gluteus maximus.

MRC scores for the iliopsoas and GM were primarily evaluated. The results of these scores were classified into four categories as shown in Figure 2. Category 1 was not included in this study, and Categories 2 to 4 comprised the present study population. Categories 3 and 4 corresponded to the “weak GM” sign (Figure 1c). Category 2 corresponded to the “weak iliopsoas with normal GM” sign (Figure 1d), and this was the complement of the “weak GM” sign since either iliopsoas or GM had to be weak for inclusion in this study. The incidences of these two signs among FW and SW groups were used as primary indicators, and statistically compared between the two groups using the chi‐square test for independence.

## 3 RESULTS

### 3.1 Enrolled patients

**FIGURE 3:**
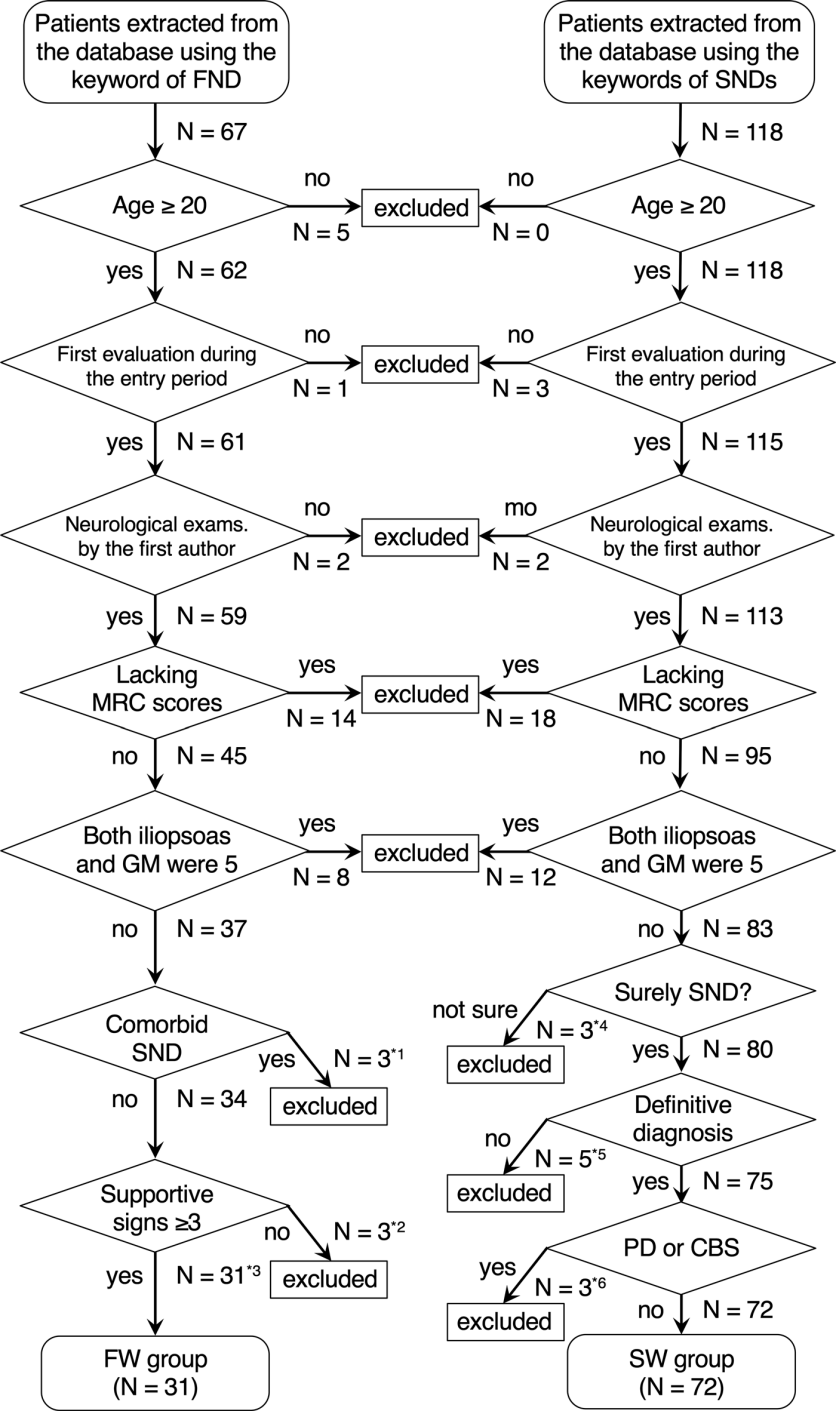
Flowchart of the enrollment of the patients. ^*1^Patients with functional overlay were excluded only when the comorbid SW may well cause lower‐limb weakness. The causes were two neuropathies and one lumbar spinal stenosis. ^*2^All three patients had two supportive signs. ^*3^Out of seven supportive signs, eight patients showed three signs, 11 showed four, six showed five, and six showed six. ^*4^For these three patients recruited as SND, we could not determine whether the final diagnosis was FND or SND, partly because of prompt recovery. ^*5^We were sure that these patients had SND based on clinical signs and ancillary tests, but we could not determine the definitive diagnosis. ^*6^Two patients with PD and a patient with CBS. CBS, corticobasal syndrome; FND, functional neurological disorders; FW, functional weakness; GM, gluteus maximus; MRC, Medical Research Council; PD, Parkinson's disease; SND, structural neurological disorders; SW, structural weakness.

Patient records first extracted from the database were 67 in the FND group and 118 in the SND group. The flowchart of the enrollment is presented in Figure 3. Consequently, 31 patients with FW (15 men and 16 women, age 47.0 ± 14.6 years, range 22−74) and 72 patients with SW (45 men and 27 women, age 68.0 ± 12.4 years, range 32−88) were included. The patient was not conscious of the lower‐limb weakness in four patients with FW. The distribution of weakness in the FW group and the causative disorders in the SW group are summarized in Table 1. Individual supportive signs observed in the FW group are summarized in Table 2. All the eight patients with a negative Hoover test showed symmetrical weakness of hip flexors and extensors. The Babinski sign was negative for all 31 patients with FW.

**TABLE 1:** Characteristics of enrolled patients.

FW (*n* = 31)	
Monoparesis	9
Hemiparesis	7
Paraparesis	6
Tetraparesis	9
SW (*n* = 72)	
Stroke	7
Motor neuron diseases (ALS 29, SBMA 1, BMALL 1)	31
Spinal cord disorders (CSM 6, injury 2, tumor 1, AAD 1, MS 1, HAM 1, dural AVF 1, subacute combined degeneration 1, IVL 1)	15
Lumbar root disorder (LSS)	2
Plexopathies (radiation 1, LRPN 1)	2
Peripheral neuropathies (GBS 2, CIDP 1)	3
Neuromuscular junction disorder (LEMS)	1
Myopathies (PM/DM 4, IBM 3, myotonic dystrophy 2, congenital myopathy 1, endocrine myopathy 1)	11

Abbreviations: AAD, atlanto‐axial dislocation; ALS, amyotrophic lateral sclerosis; AVF, arteriovenous fistula; BMALL, benign monomelic atrophy of the lower limb; CIDP, chronic inflammatory demyelinating polyneuropathy; CSM, cervical spondylotic myelopathy; FW, functional weakness; GBS, Guillain–Barré syndrome; HAM, HTLV‐1 associated myelopathy; IBM, inclusion body myositis; IVL, intravascular lymphoma; LEMS, Lambert–Eaton myasthenic syndrome; LRPN, lumbosacral radiuloplexus neuropathy; LSS, lumbar spinal stenosis; MS, multiple sclerosis; PM/DM, polymyositis/dermatomyositis; SBMA, spinal and bulbar muscular atrophy; SW, structural weakness.

**TABLE 2:** Supportive signs observed in the functional weakness (FW) group (*n* = 31).

Functional pattern in the Hoover test	23
Functional pattern in the Sonoo abductor test	22
Paradoxical wrist flexion	22
Give‐way weakness	27
Normal and symmetrical reflexes^a^	10
Other positive signs	10
Psychogenic Romberg (Lempert et al., 1991)	2
Dragging of the leg (Lempert et al., 1991)	1
Longer contact time of the paretic leg	1
“Paradoxical quadriceps weakness”^b^	6
Normal EMG^*^ ^c^	20

^a^ Normal and symmetrical reflexes were adopted as a supportive sign only when the corresponding muscles were asymmetrically weak.

^b^ “Paradoxical quadriceps weakness” was judged to be positive when, during the MRC score examination of the knee extensor force, the knee‐extended position was broken by the examiner's force, whereas the knee‐flexed position was maintained without break, similar to the paradoxical wrist flexion (Sonoo, 2020).

^c^ Normal recruitment with poor activation and no denervation potentials in needle EMG of a weak muscle.

### 3.2 Results for the new signs

The main results are summarized in Table 3. The “weak GM” sign showed 100% sensitivity and 85% specificity for diagnosing FW. Consequently, the “weak iliopsoas with normal GM” sign, the complement of the “weak GM” sign, was 100% specific for SW. Both the iliopsoas and GM muscles were weak in 11 patients with SW: four out of 29 patients with ALS, and one patient with stroke, radiation plexopathy, Guillain–Barré syndrome, Lambert–Eaton myasthenic syndrome (LEMS), inclusion body myositis, myotonic dystrophy, and congenital myopathy, respectively. Accordingly, the “weak iliopsoas with normal GM” sign was observed in 21/22 patients with stroke or spinal cord disorders, for whom the weakness was due to pyramidal tract impairment, while both the iliopsoas and GM were weak in 4/12 patients with myopathy or neuromuscular junction disorders (LEMS).

**TABLE 3:** Medical Research Council (MRC) scores of patients.

MRC score		FW (*n* = 31)	SW (*n* = 72)	
Iliopsoas	GM			
Weak GM		31 (100%)	11 (15%)	*p* < 0.0001
Five	Four or less	2 (6%)	0 (0%)	
Four or less	Four or less	29 (94%)	11 (15%)	
Weak iliopsoas with normal GM				
Four or less	Five	0 (0%)	60 (85%)	*p* < 0.0001

Abbreviations: FW, functional weakness; GM, gluteus maximus; SW, structural weakness.

## 4 DISCUSSION

### 4.1 The significance of the present signs

It is now recommended that FND should be diagnosed based on positive signs (Aybek & Perez, 2022; Daum et al., 2014; Perez et al., 2021; Stone, 2014; Stone et al., 2011; Weintraub, 1983). Here, a “positive sign” is defined as such that documents internal inconsistency or incongruity with recognized neurological or other medical disorders and therefore positively suggest the diagnosis of FND (Stone et al., 2011). In this sense, the present two signs are not “positive signs”. The weak GM sign does not indicate any motor inconsistency that cannot occur in structural weakness and can be naturally observed in any kind of structural disorders with severe impairment. However, this sign is still helpful for FW diagnosis. In this consecutive case series, the positive rate in FW was 100%, whereas that in SW was 15%. Therefore, the posttest probability that the patient has FW will obviously increase if the weak GM sign is positive.

The complementary sign, weak iliopsoas with normal GM, is further useful for the differentiation between FW and SW. This sign was 100% specific for SW. Although 100% should be discounted as discussed later, its presence strongly suggests that the weakness is structural. There are signs having similar significance. Hoover test strongly suggests SW if the synergic movement is disintegrated and the GM muscle on the unaffected side is strong when the affected limb is elevated, or that on the affected side is weak when the unaffected limb is elevated (Sonoo, 2004). However, the latter, the more popular method as mentioned later, is rather rare (Sonoo, 2004) since GM is frequently preserved in SW as documented in this study. Sonoo abductor test is more useful in this regard since the gluteus medius muscle is more frequently weak in SND (Sonoo, 2004). The disintegration of the synergic movement with the constant weakness of the gluteus medius muscle on the affected side strongly suggests a structural weakness (Sonoo, 2004). For the paradoxical wrist flexion test, “non‐paradoxical” finding named “organic sign” was 100% specific for the structural weakness (Sonoo, 2020). These signs, as well as the present “weak iliopsoas with normal GM” sign, are a “positive sign” for SW that can contradict FW. We may need another term for this kind of “positive sign for SND,” although many conventional neurological signs may belong to this category, such as positive Babinski sign. At all events, these signs are useful in differentiating between FW and SW.

### 4.2 Relationship with the Hoover test and the global pattern of weakness

Hoover test may seem to be apparently similar to the present signs and their relationship is discussed here. As explained in Section 1, the Hoover test evaluates a synergic movement between iliopsoas and the contralateral GM. It does not intend to assess the isolated force of the GM or iliopsoas. In fact, estimation of the force of the voluntary pressing of the lower limb is necessary for the Hoover test in order to compare this with the synergic (unconscious) force of the same action (Hoover, 1908). In a modified method that is now widely employed (R. D. Adams & Victor, 1997; Diukova et al., 2013; Stone & Sharpe, 2001; Stone et al., 2002), downward pressing of the paretic leg is ordered first. Next, the examiner asks the patient to lift the unaffected leg consecutively and evaluates the increase of the downward pressure felt by the examiner's hand under the heel of the affected leg. The first part of this procedure corresponds to an MMT examination of GM in the supine position. However, such a consecutive procedure was not described in the original article of Hoover (Hoover, 1908), and a possible pitfall of this method has been suggested (Sonoo, 2004; Tremolizzo et al., 2014). At all events, the present sign simply examines the voluntary forces of the GM and iliopsoas on one side, without comparing with the synergic force during the elevation of the other leg, and therefore is applicable even when the weakness is symmetrical, in contrast to the Hoover test or Sonoo abductor test that requires an asymmetry of the weakness. In fact, Hoover teat was not diagnostic in 8/31 patients with FW presenting with symmetrical weakness. We can say that the frequent weakness of GM in FW documented in the present study is the reason why Hoover test is useful in many patients with FW.

Another sign that has more closely related with the present signs is the “global pattern of weakness”, which is sometimes included in the list of positive signs (Daum et al., 2014; Stone, 2014). This sign means that extensors and flexors are equally affected in functional weakness, in contrast to the pyramidal weakness where flexors are weaker than extensors, as had been classically believed (Lance & McLeod, 1981; Wernicke, 1889). Since weak GM in FW accompanied iliopsoas weakness in most cases in the present study, our findings directly correspond to the global pattern regarding the hip joint. The reason why we specifically focused on weak GM is that GM was much more frequently weak than other anti‐gravity muscles in FW. For example, the quadriceps femoris muscle was weak only in 10/28 patients with FW in this series for whom the MRC scores of this muscle were recorded.

### 4.3 Supposed mechanism of the weak GM sign

The mechanism whereby GM becomes weak in FW would be similar to that which we speculated for the paradoxical wrist flexion sign (Sonoo, 2020). An “active” movement, for example, wrist flexion in the flexed position, in which the patient feels that they are actively exerting a specific action with an effort, is preferentially affected in FW. The downward pressing of the whole lower limb to the bed in the supine position would be felt as a typical example of such an active movement for patients with FW, followed by the hip flexion in the same position that was weak in 94% of patients with FW.

This may be related to the findings in patients with movement disorders who were excluded from this study. In fact, there were a patient with PD and a patient with CBS manifesting the weak GM sign during the entry period. Both iliopsoas and GM were weak in the patient with PD, whereas iliopsoas was normal but GM was weak in the patient with CBS. The patient with PD might be different from those in previous reports of FND associated with PD: the patient presented with rather severe PD, in contrast to the previous reports stating that FND more commonly complicates mild stage PD or even precedes its onset (Onofrj et al., 2011; Wissel et al., 2018). This may be better explained by an automatic‐voluntary dissociation postulated for the reason of positive Hoover test in CBS (Ercoli & Stone, 2020), that is, active voluntary effort to press down the leg may be impaired in such a severe movement disorder.

### 4.4 Weak iliopsoas with normal GM in structural weakness

Weak iliopsoas with normal GM was observed in most patients (85%) with SW. It is noticeable that this sign was observed in 20/21 patients with stroke or spinal cord disorders, for whom the weakness was exhibited as a pyramidal sign. This coincides with the classical descriptions that the pyramidal weakness affects flexor muscles more than extensor muscles in the lower limbs (Lance & McLeod, 1981; Wernicke, 1889). However, several studies doubted this conventional view (R. W. Adams et al., 1990; Colebatch & Gandevia, 1989), and the apparent flexor weakness in a pyramidal sign may be only due to the inherent difference of the strength of relevant muscles (Wiles, 2017). If this is the case, the force of GM would tend to be preserved in many neurological disorders other than pyramidal weakness, which may well correspond to the frequent “weak iliopsoas with normal GM” in SW in this study. It is noteworthy that GM was constantly weak in FW despite such an inherent stronger force. Hence, the overall utility of the “weak iliopsoas with normal GM” sign would not be undermined even if the flexor weakness is not specific for the pyramidal sign. Myopathies including neuromuscular junction disorders (LEMS) rather frequently presented with weak GM. It is known that the GM is involved very early on in dystrophinopathy (Tasca et al., 2012). In this way, the high specificity of the weak GM sign obviously depends on the spectrum of the structural disorders: if myopathies are major diseases to be differentiated, the weak GM sign would not be useful for suspecting FW. This is an obvious limitation of this study. However, the subjects of this study were consecutive patients referred to the first author in university hospitals or other core city hospitals, and the documented specificity would be of reference to doctors working in similar situations.

### 4.5 Limitations of this study

There are several other limitations, or notes, to be mentioned in this study. First, the examiner was not blinded to the diagnosis of the patient. The diagnosis of FW was confirmed by three or more supportive signs other than the weak GM sign itself, but there remains the possibility that the diagnosis of FW suspected from other signs might have influenced the evaluation of the strengths of the iliopsoas and GM. Although the first author is an expert in muscle force evaluation (Sonoo, 2018) and tried to be as unbiased as possible, the unblinded design is a definite limitation of this study. Furthermore, interrater reproducibility was not tested in this study. The MRC scale is a subjective measure, and considerable interrater variation is inevitable. Investigation of the interrater reproducibility, ideally by a blinded design, is awaited.

Second limitation might be a selection bias. We did not include patients with uncertain final diagnosis or those with both functional weakness and structural disorders that may well cause a lower‐limb weakness. The excellent results were obtained in this rather “pure” population and may not be generalized to the whole population in the real world.

Considering the above two limitations, the 100% sensitivity of the weak GM sign for FW, that is, 100% specificity of the “weak Ip with normal GM” sign for SW documented in this study, might be discounted. We should not make a diagnosis solely based on a single sign (Stone, Carson, et al., 2010) but consider the whole clinical context and other neurological signs.

Third, patients in the structural group were significantly older than those in the functional group. This might be considered a drawback if the iliopsoas muscle becomes frequently weak in normal aged subjects. However, our experiences tell that our test position for the iliopsoas muscle is not broken even in aged control subjects (Sonoo, 2018), and we therefore think that the “weak Ip with normal GM” sign reflects pathological weakness and not the result of aging.

Lastly, the default maneuver of examining the force of the GM would be the test in the prone position because the effect of gravity can be evaluated by this method (Hislop et al., 2014; Kendall et al., 2005). The reason why we used the spine test needs to be explained. One drawback of the prone test is that the test position might be broken even in normal subjects since the hyperextension of the hip‐joint is a non‐physiological movement (Sonoo, 2018). The supine test is mentioned by some textbooks (Guarantors of Brain, 2010; Hislop et al., 2014) and comprises a part of the Hoover test as already mentioned. Our method is different from the supine test in past literature in two points. First, we did not place the examiner's hand under the heel but under the knee, as proposed by other authors for the Hoover test in the sitting position (Espay et al., 2018; Stone, 2014; Stone, 2009). Placing the examiner's hand under the knee adheres to the principle in the MRC examination that one joint should be tested when possible, but the low sensitivity to detect weakness due to the shorter lever would be a disadvantage. Second, we conducted the fixation of the proximal part by placing the examiner's other hand over the pelvis, which may partially ameliorate the low sensitivity mentioned above.

The new signs described in this study can be easily evaluated using only MRC examinations. It will likely be helpful in judging whether the weakness is functional or structural from the bedside neurological examinations in the general neurology setting.

## AUTHOR CONTRIBUTIONS

Masahiro Sonoo: Conceptualization (lead); data curation (lead); formal analysis (lead); funding acquisition (lead); investigation (lead); methodology (lead); project administration (lead); resources (lead); visualization (lead); writing—original draft preparation (lead). Takamichi Kanbayashi: Project administration (supporting); resources (supporting); writing—review and editing (supporting). Shunsuke Kobayashi: Conceptualization (supporting); writing—review and editing (supporting). Hiromasa Matsuno, Takahiro Nakayama, Ichiro Imafuku, Tetsuo Ando, and Toshio Fukutake: Resources (supporting); writing—review and editing (supporting).

## CONFLICT OF INTEREST STATEMENT

The authors declare no conflict of interest.

### PEER REVIEW

The peer review history for this article is available at https://publons.com/publon/10.1002/brb3.3135.

## Data Availability

The data that support the findings of this study are available from the corresponding author upon reasonable request.

## References

[brb33135-bib-0002] Adams, R. D. , & Victor, M. (1997). Principle of neurology (6th ed.). McGraw‐Hill.

[brb33135-bib-0003] Adams, R. W. , Gandevia, S. C. , & Skuse, N. F. (1990). The distribution of muscle weakness in upper motorneuron lesions affecting the lower limb. Brain, 113, 1459–1476. 10.1093/brain/113.5.1459 2245306

[brb33135-bib-0004] American Psychiatric Association . (2013). Diagnostic and statistical manual of mental disorders (5th ed.). American Psychiatric Press Inc.

[brb33135-bib-0005] Aybek, S. , & Perez, D. L. (2022). Diagnosis and management of functional neurological disorder. BMJ, 376, o64. 10.1136/bmj.o64 35074803

[brb33135-bib-0001] Babinski, J. (1967). The differential diagnosis of organic hemiplegia and hysteric hemiplegia: 1900. Translated by Mannen H and Imamura M. Shinkei Kenkyu No Shimpo. Advances in Neurological Sciences, 11, 664–674. 4873147

[brb33135-bib-0006] Chabrol, H. , Peresson, G. , & Clanet, M. (1995). Lack of specificity of the traditional criteria for conversion disorders. European Psychiatry, 10, 317–319. 10.1016/0924-9338(96)80314-2 19698360

[brb33135-bib-0007] Colebatch, J. G. , & Gandevia, S. C. (1989). The distribution of muscular weakness in upper motor neuron lesions affecting the arm. Brain, 112, 749–763. 10.1093/brain/112.3.749 2731028

[brb33135-bib-0008] Daum, C. , Gheorghita, F. , Spatola, M. , Stojanova, V. , Medlin, F. , Vingerhoets, F. , Berney, A. , Gholam‐Rezaee, M. , Maccaferri, G. E. , Hubschmid, M. , & Aybek, S. (2015). Interobserver agreement and validity of bedside ‘positive signs’ for functional weakness, sensory and gait disorders in conversion disorder: A pilot study. Journal of Neurology, Neurosurgery, and Psychiatry, 86, 425–430. 10.1136/jnnp-2013-307381 24994927

[brb33135-bib-0009] Daum, C. , Hubschmid, M. , & Aybek, S. (2014). The value of ‘positive’ clinical signs for weakness, sensory and gait disorders in conversion disorder: A systematic and narrative review. Journal of Neurology, Neurosurgery, and Psychiatry, 85, 180–190. 10.1136/jnnp-2012-304607 23467417

[brb33135-bib-0010] Diukova, G. M. , Ljachovetckaja, N. I. , Begljarova, M. A. , & Gavrileyko, G. I. (2013). Simple quantitative analysis of Hoover's test in patients with psychogenic and organic limb pareses. Journal of Psychosomatic Research, 74, 361–364. 10.1016/j.jpsychores.2012.10.004 23497840

[brb33135-bib-0011] Ercoli, T. , & Stone, J. (2020). False positive Hoover's sign in apraxia. Movement Disorders Clinical Practice, 7, 567–568. 10.1002/mdc3.12970 32626806 PMC7328420

[brb33135-bib-0012] Espay, A. J. , Aybek, S. , Carson, A. , Edwards, M. J. , Goldstein, L. H. , Hallett, M. , Lafaver, K. , Lafrance, W. C. , Lang, A. E. , Nicholson, T. , Nielsen, G. , Reuber, M. , Voon, V. , Stone, J. , & Morgante, F. (2018). Current concepts in diagnosis and treatment of functional neurological disorders. JAMA Neurology, 75, 1132–1141. 10.1001/jamaneurol.2018.1264 29868890 PMC7293766

[brb33135-bib-0014] Gould, R. , Miller, B. L. , Goldberg, M. A. , & Benson, D. F. (1986). The validity of hysterical signs and symptoms. Journal of Nervous and Mental Disease, 174, 593–597. 10.1097/00005053-198610000-00003 3760849

[brb33135-bib-0015] Guarantors of Brain . (2010). Aids to the examination of the peripheral nervous system (5th ed.). Saunders Elsevier.

[brb33135-bib-0016] Hallett, M. (2018). Patients with Parkinson disease are prone to functional neurological disorders. Journal of Neurology, Neurosurgery, and Psychiatry, 89, 557. 10.1136/jnnp-2017-317684 29549188 PMC5970010

[brb33135-bib-0017] Hallett, M. , Aybek, S. , Dworetzky, B. A. , Mcwhirter, L. , Staab, J. P. , & Stone, J. (2022). Functional neurological disorder: New subtypes and shared mechanisms. Lancet Neurology, 21, 537–550. 10.1016/S1474-4422(21)00422-1 35430029 PMC9107510

[brb33135-bib-0018] Hislop, H. J. , Avers, D. , & Brown, M. (2014). Daniels and Worthingham's Muscle testing: Techniques of manual examination and performance testing (9th ed.). W. B. Saunders.

[brb33135-bib-0019] Hoover, C. F. (1908). A new sign for the detection of malingering and functional paresis of the lower extremities. JAMA, LI, 746–747. 10.1001/jama.1908.25410090028001h

[brb33135-bib-0020] Hu, H. , Meijer, O. G. , Van Dieën, J. H. , Hodges, P. W. , Bruijn, S. M. , Strijers, R. L. , Nanayakkara, P. W. B. , Van Royen, B. J. , Wu, W. H. , & Xia, C. (2011). Is the psoas a hip flexor in the active straight leg raise? European Spine Journal, 20, 759–765. 10.1007/s00586-010-1508-5 20625774 PMC3082678

[brb33135-bib-0021] Kendall, F. P. , McCreary, E. K. , Provance, P. G. , Rodgers, M. M. , & Romani, W. A. (2005).Muscles: Testing and function with posture and pain (5th ed.). Lippincott Williams & Wilkins.

[brb33135-bib-0022] Lance, J. W. , & McLeod, J. G. (1981). A physiological approach to clinical neurology. Butterworth.

[brb33135-bib-0023] Lanska, D. (2006). Functional weakness and sensory loss. Seminars in Neurology, 26, 297–309. 10.1055/s-2006-945516 16791776

[brb33135-bib-0024] Lempert, T. , Brandt, T. , Dieterich, M. , & Huppert, D. (1991). How to identify psychogenic disorders of stance and gait. A video study in 37 patients. Journal of Neurology, 238, 140–146. 10.1007/BF00319680 1869889

[brb33135-bib-0025] Magee, K. R. (1962). Hysterical hemiplegia and hemianesthesia. Postgraduate Medicine, 31, 339–345. 10.1080/00325481.1962.11694610 14468245

[brb33135-bib-0026] Onofrj, M. , Thomas, A. , Tiraboschi, P. , Wenning, G. , Gambi, F. , Sepede, G. , Di Giannantonio, M. , Di Carmine, C. , Monaco, D. , Maruotti, V. , Ciccocioppo, F. , D'amico, M. C. , & Bonanni, L. (2011). Updates on somatoform disorders (SFMD) in Parkinson's disease and dementia with Lewy bodies and discussion of phenomenology. Journal of the Neurological Sciences, 310, 166–171. 10.1016/j.jns.2011.07.010 21813140

[brb33135-bib-0027] Perez, D. L. , Edwards, M. J. , Nielsen, G. , Kozlowska, K. , Hallett, M. , & Lafrance, W. C., Jr. (2021). Decade of progress in motor functional neurological disorder: Continuing the momentum. Journal of Neurology, Neurosurgery, and Psychiatry, 92, 668–677. 10.1136/jnnp-2020-323953 PMC8440656 33722822

[brb33135-bib-0028] Sonoo, M. (2004). Abductor sign: A new reliable sign to detect unilateral nonorganic paresis of the lower limb. Journal of Neurology, Neurosurgery, and Psychiatry, 75, 121–125. 14707320 PMC1757483

[brb33135-bib-0029] Sonoo, M. (2018).Guidebook for MMT and needle EMG. Chugai Igakusya.

[brb33135-bib-0030] Sonoo, M. (2020). Paradoxical wrist flexion: A new test to detect functional weakness of the upper limb. eNeurologicalSci, 22, 100302. 10.1016/j.ensci.2020.100302 33344786 PMC7735966

[brb33135-bib-0031] Sonoo, M. (2023). Borderline regions between neurology and psychiatry, focusing particularly on the functional neurological disorders. Rinsho Shinkeigaku. Clinical Neurology, 63, 135–144. 10.5692/clinicalneurol.cn-001817 36843086

[brb33135-bib-0032] Stone, J. (2002). Functional weakness and sensory disturbance. Journal of Neurology, Neurosurgery, and Psychiatry, 73, 241–245. 10.1136/jnnp.73.3.241 12185152 PMC1738014

[brb33135-bib-0033] Stone, J. (2009). The bare essentials: Functional symptoms in neurology. Practical Neurology, 9, 179–189. 10.1136/jnnp.2009.177204 19448064

[brb33135-bib-0034] Stone, J. (2014). Functional neurological disorders: The neurological assessment as treatment. Neurophysiologie Clinique = Clinical Neurophysiology, 44, 363–373. 10.1016/j.neucli.2014.01.002 25306077

[brb33135-bib-0035] Stone, J. , Carson, A. , Duncan, R. , Roberts, R. , Warlow, C. , Hibberd, C. , Coleman, R. , Cull, R. , Murray, G. , Pelosi, A. , Cavanagh, J. , Matthews, K. , Goldbeck, R. , Smyth, R. , Walker, J. , & Sharpe, M. (2010). Who is referred to neurology clinics?—The diagnoses made in 3781 new patients. Clinical Neurology and Neurosurgery, 112, 747–751. 10.1016/j.clineuro.2010.05.011 20646830

[brb33135-bib-0036] Stone, J. , Lafrance, W. C., Jr. , Brown, R. , Spiegel, D. , Levenson, J. L. , & Sharpe, M. (2011). Conversion disorder: Current problems and potential solutions for DSM‐5. Journal of Psychosomatic Research, 71, 369–376. 10.1016/j.jpsychores.2011.07.005 22118377

[brb33135-bib-0037] Stone, J. , & Sharpe, M. (2001). Hoover's sign. Practical Neurology, 1, 50–53. 10.1046/j.1474-7766.2001.00607.x

[brb33135-bib-0038] Stone, J. , Warlow, C. , & Sharpe, M. (2010). The symptom of functional weakness: A controlled study of 107 patients. Brain, 133, 1537–1551. 10.1093/brain/awq068 20395262

[brb33135-bib-0039] Swash, M. (2012). Why are upper motor neuron signs difficult to elicit in amyotrophic lateral sclerosis? Journal of Neurology, Neurosurgery, and Psychiatry, 83, 659–662. 10.1136/jnnp-2012-302315 22496581

[brb33135-bib-0040] Tasca, G. , Iannaccone, E. , Monforte, M. , Masciullo, M. , Bianco, F. , Laschena, F. , Ottaviani, P. , Pelliccioni, M. , Pane, M. , Mercuri, E. , & Ricci, E. (2012). Muscle MRI in Becker muscular dystrophy. Neuromuscular Disorders, 22(Suppl 2), S100–S106. 10.1016/j.nmd.2012.05.015 22980760

[brb33135-bib-0041] Tremolizzo, L. , Susani, E. , Riva, M. A. , Cesana, G. , Ferrarese, C. , & Appollonio, I. (2014). Positive signs of functional weakness. Journal of the Neurological Sciences, 340, 13–18. 10.1016/j.jns.2014.03.003 24656598

[brb33135-bib-0042] Weintraub, M. I. (1983). Hysterical conversion reactions: A clinical guide to diagnosis and treatment. Spectrum Publications.

[brb33135-bib-0043] Wernicke, C. (1889). Zur Kenntniss der cerebralen Hemiplegie. Berliner Klinische Wochenschrift, 26, 969–970.

[brb33135-bib-0044] Wiles, C. M. (2017). Pyramidal weakness. Practical Neurology, 17, 241–242. 10.1136/practneurol-2016-001584 28119376

[brb33135-bib-0045] Wissel, B. D. , Dwivedi, A. K. , Merola, A. , Chin, D. , Jacob, C. , Duker, A. P. , Vaughan, J. E. , Lovera, L. , Lafaver, K. , Levy, A. , Lang, A. E. , Morgante, F. , Nirenberg, M. J. , Stephen, C. , Sharma, N. , Romagnolo, A. , Lopiano, L. , Balint, B. , Yu, X. X. , … Espay, A. J. (2018). Functional neurological disorders in Parkinson disease. Journal of Neurology, Neurosurgery, and Psychiatry, 89, 566–571. 10.1136/jnnp-2017-317378 29549192

